# Supplementary material for: Novel Driver Strength Index highlights important cancer genes in TCGA PanCanAtlas patients
Source: PeerJ. 2022 Aug 11;10:e13860. doi: 10.7717/peerj.13860 (PMC9375969; doi:10.7717/peerj.13860)
Supplement: Supplemental Information 2 [file peerj-10-13860-s002.zip › COHORTS/genes plots/age/2021_8_16_14_9_distribution_cohorts_females_35-39.pdf]

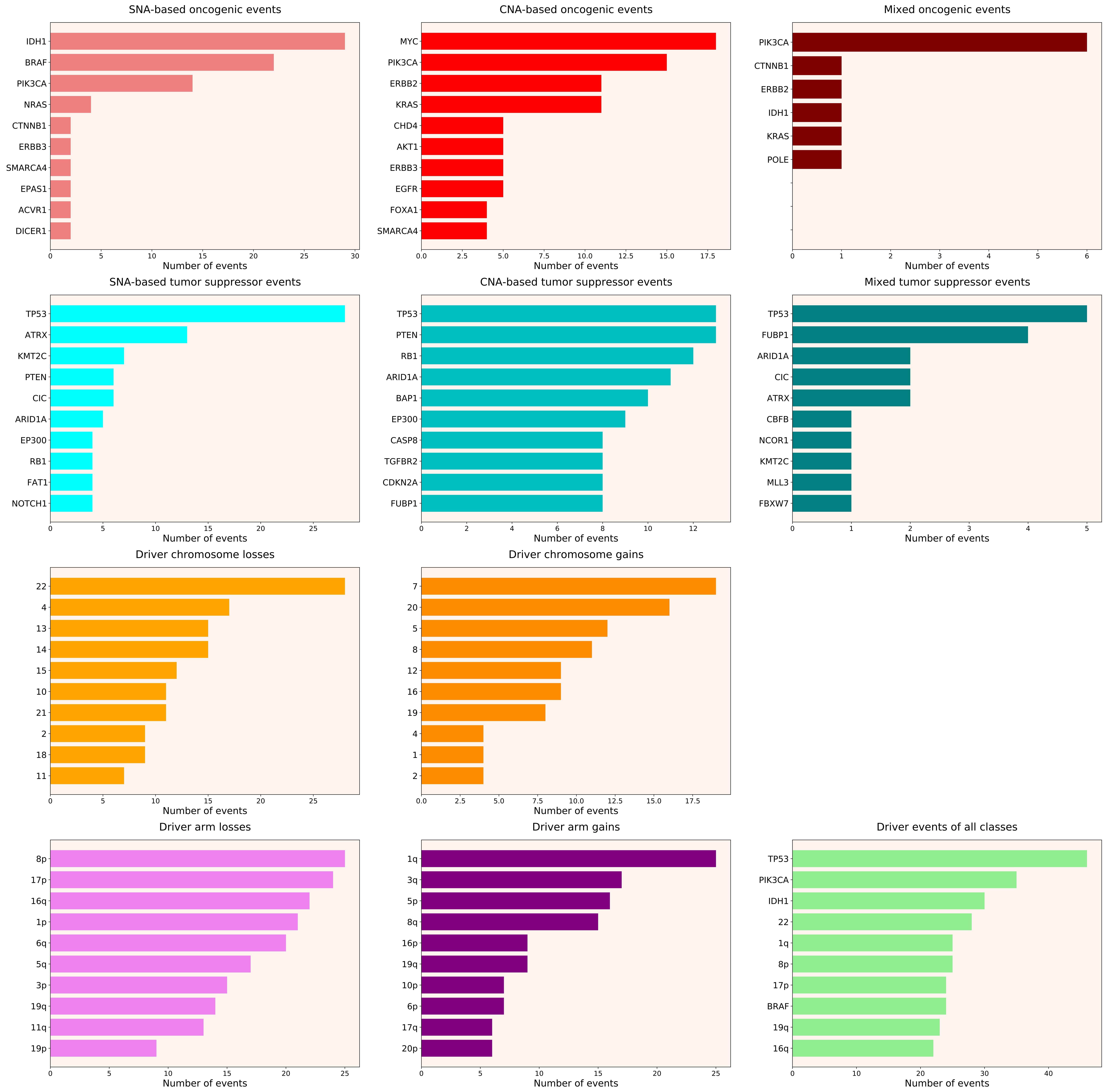
SNA-based oncogenic events
IDH1
BRAF
PIK3CA
NRAS
CTNNB1
ERBB3
SMARCA4
EPAS1
ACVR1
DICER1
0 5 10 15 20 25 30
Number of events
CNA-based oncogenic events
MYC
PIK3CA
ERBB2
KRAS
CHD4
AKT1
ERBB3
EGFR
FOXA1
SMARCA4
0.0 2.5 5.0 7.5 10.0 12.5 15.0 17.5
Number of events
Mixed oncogenic events
PIK3CA
CTNNB1
ERBB2
IDH1
KRAS
POLE
0 1 2 3 4 5 6
Number of events
SNA-based tumor suppressor events
TP53
ATRX
KMT2C
PTEN
CIC
ARID1A
EP300
RB1
FAT1
NOTCH1
0 5 10 15 20 25
Number of events
CNA-based tumor suppressor events
TP53
PTEN
RB1
ARID1A
BAP1
EP300
CASP8
TGFBR2
CDKN2A
FUBP1
0 2 4 6 8 10 12
Number of events
Mixed tumor suppressor events
TP53
FUBP1
ARID1A
CIC
ATRX
CBFB
NCOR1
KMT2C
MLL3
FBXW7
0 1 2 3 4 5
Number of events
Driver chromosome losses
22
4
13
14
15
10
21
2
18
11
0 5 10 15 20 25
Number of events
Driver chromosome gains
7
20
5
8
12
16
19
4
1
2
0.0 2.5 5.0 7.5 10.0 12.5 15.0 17.5
Number of events
Driver arm losses
8p
17p
16q
1p
6q
5q
3p
19q
11q
19p
0 5 10 15 20 25
Number of events
Driver arm gains
1q
3q
5p
8q
16p
19q
10p
6p
17q
20p
0 5 10 15 20 25
Number of events
Driver events of all classes
TP53
PIK3CA
IDH1
22
1q
8p
17p
BRAF
19q
16q
0 10 20 30 40
Number of events
